# Elevated plasma total homocysteine levels are associated with behavioral and psychological symptoms in dementia with Lewy bodies

**DOI:** 10.3389/fnins.2024.1406694

**Published:** 2024-11-19

**Authors:** Guili Zhang, Shuai Liu, Ying Xu, Ling-Yun Ma, Wei Zhang, Yong Ji

**Affiliations:** ^1^Department of Neurology, Beijing Tiantan Hospital, Capital Medical University, Beijing, China; ^2^China National Clinical Research Center for Neurological Diseases, Beijing, China; ^3^Tianjin Key Laboratory of Cerebrovascular and Neurodegenerative Diseases, Tianjin, China; ^4^Department of Neurology, Tianjin Dementia Institute, Tianjin Huanhu Hospital, Tianjin, China; ^5^Department of Neurology, Fuxing Hospital, Capital Medical University, Beijing, China

**Keywords:** dementia with Lewy bodies, homocysteine, behavioral and psychological symptoms of dementia, cognitive dysfunction, dementia

## Abstract

**Objective:**

To investigate the association between plasma total homocysteine (tHcy) levels and behavioral and psychological symptoms of dementia (BPSD) in dementia with Lewy bodies (DLB) patients.

**Methods:**

A total of 82 DLB patients and 134 age-matched healthy controls were included in this study. DLB patients were assessed using the Mini-Mental Status Examination (MMSE), the Clinical Dementia Rating Scale (CDR), and the Neuropsychiatric Inventory (NPI). Plasma tHcy, serum vitamin B12, and folate levels were measured in all study participants. We used Spearman’s rank correlation test to analyze the association between tHcy concentrations and NPI scores, MMSE, CDR, and the duration of dementia in DLB patients.

**Results:**

Clinically significant BPSD was present in 92.7% of DLB patients. The most frequent BPSD were hallucinations (30.4%), apathy (30.4%), and delusions (26.8%). Elevated plasma tHcy levels were significantly associated with total NPI scores in DLB patients, particularly in 10 NPI sub-domains, except for agitation/aggression and disinhibition. No statistically significant association was found between plasma tHcy levels and MMSE, CDR, or dementia duration.

**Limitations:**

Longitudinal studies with larger sample sizes are required to further explore the relationship between tHcy levels and BPSD in DLB patients as the disease progresses.

**Conclusion:**

Our study highlighted the high incidence of BPSD and was the first to show that BPSD is associated with elevated plasma tHcy levels in DLB patients in China. These results support the hypothesis that controlling homocysteine levels could offer a new direction for managing BPSD.

## 1 Introduction

Dementia with Lewy bodies (DLB), as a neurocognitive disorder, is the second most common cause of neurodegenerative dementia after Alzheimer’s disease (AD), accounting for 7.5% or more of all dementias (Walker et al., 2015).

The cognitive symptoms, along with varying combinations of core clinical features and the high incidence of early prominent behavioral and psychological symptoms of dementia (BPSD), are clinical features of DLB. The cognitive pattern of DLB typically shows prominent deficits in executive function and visual processing, whereas verbal episodic memory and naming abilities are relatively preserved (Kim and Lee, 2014; Monji et al., 2005; Reif et al., 2005; Seshadri et al., 2002; Virtanen et al., 2005; Zheng et al., 2014). Finally, attention is also affected, with reduced sustained and divided attention abilities and increased attentional fluctuations (McKeith et al., 2017; Soni et al., 2019; Tabet et al., 2006). Additionally, a recent study by Bao et al. (2022) found that very mild DLB was associated with impairment of attentional/executive, visuospatial, visuoconstructive, and naming abilities, as well as with difficulties in retrieval of episodic memory. As DLB progresses to its mild form, further deterioration of executive function leads to worse performance on tests of inhibition, mental flexibility, and verbal initiation. The BPSD spectrum commonly includes abnormal thought, perception, mood, and behavior disorders in patients with dementia (Kales et al., 2015; Seitz et al., 2011). Behavioral and psychiatric symptoms, such as visual hallucinations, RBD, depression, anxiety, and delirium, can be present very early and before the onset of memory impairment in DLB (Morris, 1993). The most frequent BPSD in the early stages of DLB includes visual hallucinations and delusions (Ballard et al., 1999), which have been reported to occur in up to 60 and 80% of patients, respectively (McKeith, 2006; Walker et al., 2015). Current clinical criteria for both DLB and mild cognitive impairment with Lewy bodies (MCI-LB) highlight the importance of BPSD due to its high incidence, which mainly includes five neuropsychiatric supportive features: non-visual hallucinations, systematized delusions, apathy, anxiety, and depression. MCI-LB is differentiated from other causes of MCI based on the presence of core clinical features (cognitive fluctuations, recurrent visual hallucinations, REM sleep behavior disorder, and parkinsonism) associated with dementia with Lewy bodies (DLB). Furthermore, compared with AD patients, DLB patients experienced more pronounced anxiety, delusions, hallucinations, apathy, sleep disturbance, and other BPSD, even in the predementia stage, leading to faster deterioration in the quality of life early in the disease course (Donaghy et al., 2022; Irizarry et al., 2005). Numerous studies have reported that BPSD is the most challenging aspect of DLB, which affects cognitive function, resulting in excess disability, increases the burden for the caregiver, and is also the cause of repeated hospitalization, early institutionalization, and increased cost burdens (Donaghy et al., 2022; Irizarry et al., 2005; Tabata et al., 2017). Although there is no curative treatment for cognitive impairment in DLB, management and treatment of BPSD in the early stage probably delay further clinical decline, improve the quality of life of DLB patients, and reduce caregivers’ burden. Therefore, it is important to identify the treatable risk factors for BPSD in DLB, as this provides potential targets for interventions in DLB.

There are studies reporting the association between serum B12, folate, and BPSD in Alzheimer’s disease (AD) (Engelborghs et al., 2004; Quadri et al., 2004; Stuerenburg et al., 2004; Vogel et al., 2009). It has been found that B-vitamin deficiency, particularly folate and vitamin B12, has been linked to cognitive impairment (slow mentation, memory deficits, and confusion), mood changes (agitation, depression, and mania), and psychotic symptoms (paranoia, auditory, visual hallucinations and delusions) in patients with dementia. However, serum vitamin B12 and folate may not reflect the actual status of these vitamins in the brain. The homocysteine levels are considered a better test. Homocysteine, an important amino acid, is the cytotoxic product of the methionine cycle. Given the conversion of homocysteine to cysteine or methionine requires folate and vitamin B12, a deficiency of folate and vitamin B12 can cause elevated blood homocysteine levels (Vogel et al., 2009). Elevated homocysteine acts as a strong toxic substance in the body, eventually damaging the blood vessels and resulting in blood clotting or thrombus formation, which is involved in the pathogenesis of cardiovascular and cerebrovascular disorders (Fácila et al., 2005; Virtanen et al., 2005). In recent years, elevated plasma homocysteine levels have been reported to be a risk factor not only for vascular diseases, including coronary heart disease and stroke, but also associated with cognitive impairment, vascular dementia, and AD (Clarke et al., 1991; Ravaglia et al., 2005; Seshadri et al., 2002). In addition, studies have shown that elevated plasma homocysteine might be directly related to depression, schizophrenia, and other psychiatric disorders (Monji et al., 2005; Reif et al., 2005; Vogel et al., 2009). Moreover, most studies have proposed that BPSD may be correlated with elevated plasma total homocysteine (tHcy) levels in AD and vascular dementia patients (Kim and Lee, 2014; Soni et al., 2019; Zheng et al., 2014), suggesting that hyperhomocysteinemia may play a role in the pathogenesis of BPSD, although other studies found no significant association between tHcy and BPSD (Tabet et al., 2006). To the best of our knowledge, the associations of differing levels of plasma tHcy serum folate and vitamin B12 with BPSD in DLB patients remain unreported. Our previous findings indicate a significant association between elevated tHcy levels and DLB in Chinese populations, which has prompted us to investigate whether there were any associations between differing tHcy levels and BPSD in DLB patients.

In the present study, we used a case–control design to evaluate the associations of tHcy levels and BPSD of DLB patients in the Chinese population. There may be a hypothesized etiological role in DLB’s BPSD.

## 2 Methods

### 2.1 Participants

**Figure 1:**
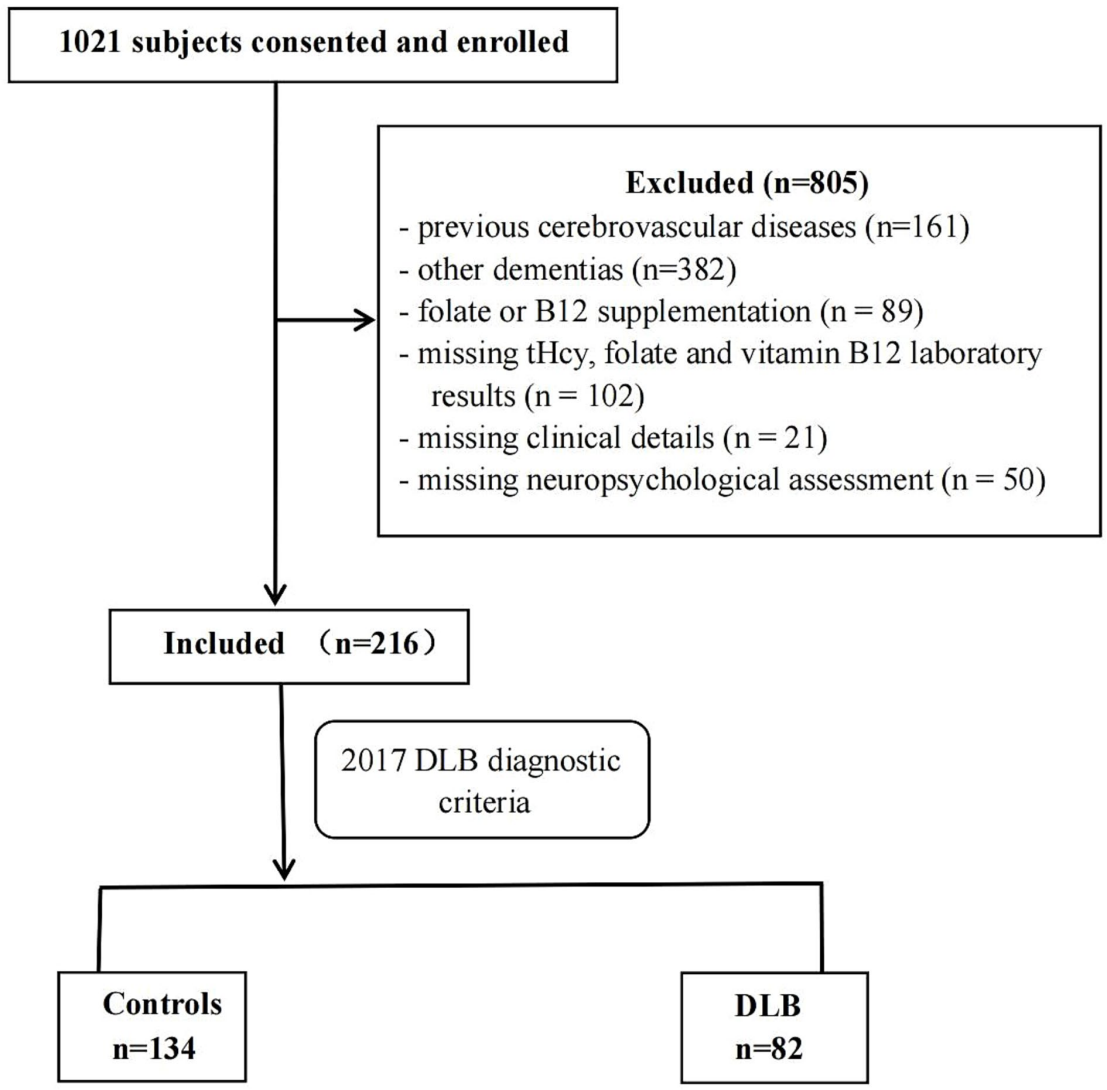
Flowchart of study subjects’ selection.

This study included a total of 216 subjects (>55 years of age): 82 DLB patients and 134 age-matched healthy controls. The recruitment, selection, and classification of subjects were performed according to the flow chart shown in Figure 1. Indeed, 40 of 82 DLB patients overlap with a part of the samples in our published previous study (Zhang et al., 2021). Between September 2017 and August 2020, a total of 1,021 participants (>55 years of age) were consecutively recruited from the neurological department at Beijing Tiantan Hospital in China, which is a center of the China Lewy Body Disease Collaborative Alliance. All participants were examined by a committee comprising at least two neurologists and one neuropsychologist. Subjects whose magnetic resonance exam showed cerebrovascular lesions (*n* = 161) or dementia patients with conditions other than DLB (*n* = 382) were excluded. Additionally, 262 individuals were excluded for the following reasons: their plasma tHcy, serum folate, and vitamin B12 concentrations were unavailable (*n* = 102); they were suffering from malnutrition and vitamin B12 deficiency; they were treated with folate or vitamin B12 in the last 3 months (*n* = 89); neuropsychological screen (*n* = 50) or their clinical details (*n* = 21) were absent. In total, the remaining 216 subjects—including 82 DLB and 134 healthy controls—constituted our study sample. Dementia was diagnosed based on the clinical criteria of the Diagnostic and Statistical Manual of Mental Disorders, 4th edition (DSM-IV) (Bao et al., 2022). Patients with probable DLB were clinically diagnosed according to McKeith’s fourth consensus report of the DLB Consortium in 2017 (Kales et al., 2015). For these patients, dementia had to start at least 1 year before the onset of the extrapyramidal syndrome (otherwise, they would be diagnosed with PD dementia) and initially present with at least two core clinical DLB features [visual hallucinations, parkinsonism, fluctuating cognition, and/or rapid eye movement sleep behavior disorder (RBD)] as shown at first presentation at our memory clinic. Furthermore, ^18^F-fluorodeoxyglucose positron emission tomography with computed tomography (^18^F-FDG-PET-CT) scans of patients showing biomarkers such as occipital hypometabolism and/or the cingulate island sign warranted a probable DLB diagnosis. Healthy controls were considered free of cognitive impairments with Mini-Mental State Examination (MMSE) scores of >26. This study was designed and conducted in accordance with the Declaration of Helsinki, and written informed consent was obtained from all participants’ legal guardians.

### 2.2 Neurological/clinical exam and neuropsychological screen

All subjects underwent a standardized diagnostic workup that included a semistructured medical history interview, collection of an informant-based history, neurological and physical examinations, a neuropsychological assessment, a brain MRI, and a standard laboratory test. The Mini-Mental State Examination (MMSE) has become the best-known and most often-used short screening tool for providing an overall measure of cognitive impairment (Folstein et al., 1975). MMSE is a 30-question assessment of cognitive function that evaluates attention and orientation, memory, registration, recall, calculation, language, and ability to draw a complex polygon. In this study, a score of 21–26 on the MMSE scale fell into the mild dementia category, while a score of 11–20 indicated moderate dementia. Clinical dementia rating (CDR) was used to assess the dementia severity, and CDR global scores (range 0–3) were recorded. The CDR is rated by the patients and caregivers of the patients, such as their spouse, child, and nurse (Lanctôt et al., 2008). CDR 1 was combined to present mild dementia, CDR 2 presented moderate dementia, and CDR 3 presented severe dementia. BPSD was assessed with the 12-item Neuropsychiatric Inventory (NPI) using information provided by the caregiver (Brown et al., 2007). The composite scores of the 12 subdomains were then added up to obtain the total NPI score (zero to 144). The patients were evaluated according to the behavioral changes displayed for the 12 subdomains, including delusion, hallucinations, agitation/aggression, depression/dysphoria, anxiety, euphoria/elation, apathy/indifference, disinhibition, irritability/lability, aberrant motor behavior, sleep/nighttime behavior, and appetite/eating changes in the 4 weeks prior to the assessment. When a specific abnormal behavior was found in one of the subdomains, a composite score (zero to 12 points) was obtained by multiplying the score for frequency (zero to four) with the score for severity (zero to three). Category scores of 4 or more indicate clinical significance (Lyketsos et al., 2002).

### 2.3 Biochemical measurement

The blood homocysteine, folate, and vitamin B12 concentrations were measured in both DLB patients and healthy controls. Participants’ blood samples were obtained following an overnight fast of 12 to 14 h. The samples were drawn by venipuncture into 5-mL plain evacuated tubes and then centrifuged at 2,000 *g* for 10 min. All specimens were collected in plastic vacuum tubes containing EDTA and analyzed within 1 h or stored at −80°C until use. Plasma tHcy, serum folate, and serum vitamin B12 concentrations were determined by electrochemiluminescence immunoassays (ECLIA) from Roche Diagnostics that were run on a COBAS 8000 e 602 analyzer (Roche Diagnostics, Switzerland).

### 2.4 Statistical analysis

Continuous variables were expressed as the mean and standard deviation, while categorical variables were expressed as proportions. To assess differences between the healthy control and DLB groups, the Mann–Whitney *U* test was used for continuous variables. The associations between the tHcy concentrations and the NPI score, MMSE, CDR, and duration of dementia were analyzed using Spearman’s rank correlation test. Statistical significance was defined as a *p-*value of <0.05. All statistical analyses were performed using SPSS 25.0 (IBM Corp., Armonk, NY, United States).

## 3 Results

### 3.1 Demographic and clinical characteristics

**Figure 2:**
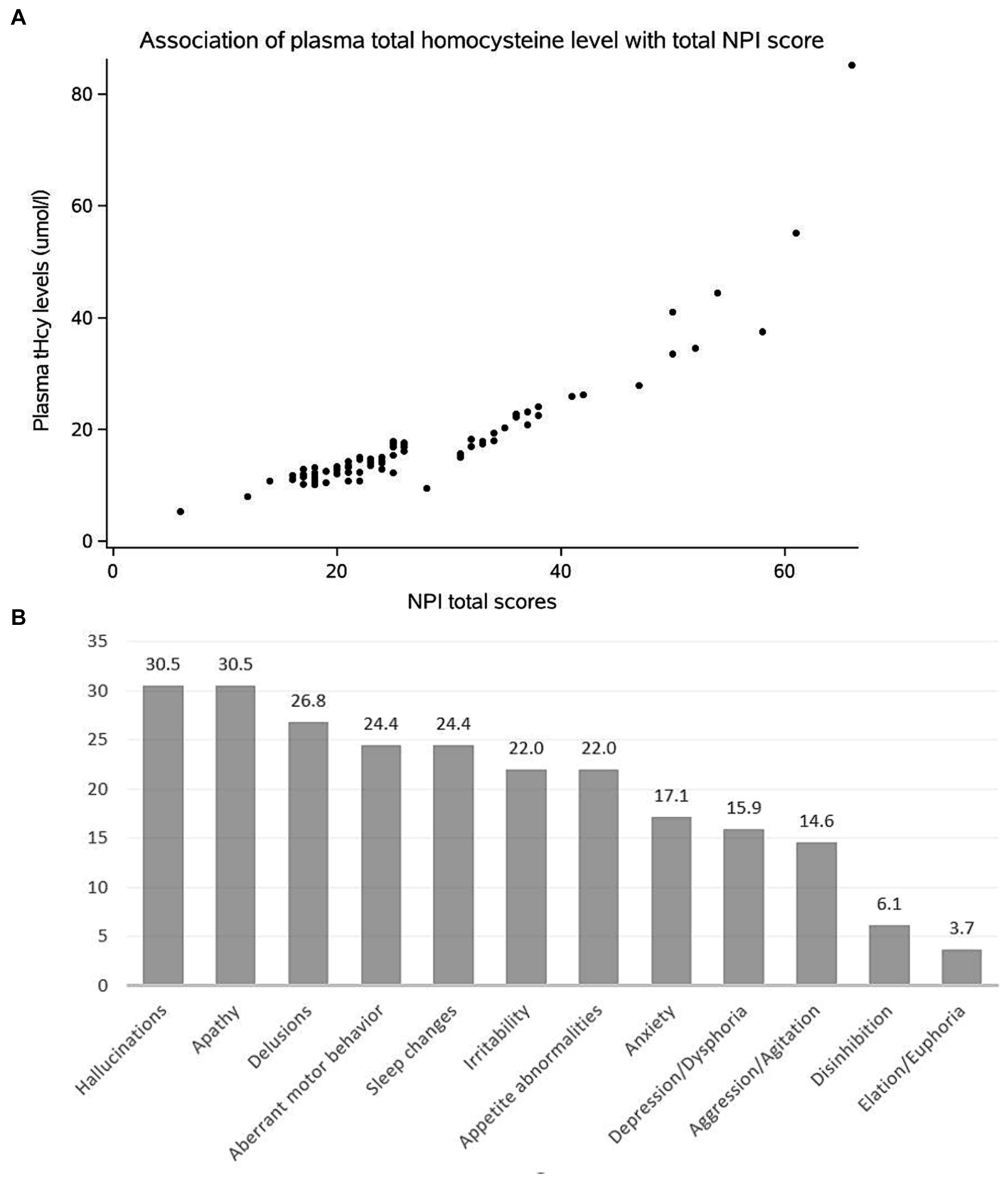
(A) Scatter diagram to show the association of the tHcy level with total NPI score in DLB patients. (B) Frequency of clinically significant BPSD in DLB patients.

The baseline characteristics of DLB patients and healthy controls in the study are shown in Table 1. The two groups were matched for gender. Compared with the control groups, DLB patients were older and had more years of education (*p* < 0.001). The MMSE scores were lower in the DLB group than in the control group (*p* < 0.001). DLB patients also had higher tHcy concentrations (17.73 ± 9.06 μmol/L) (*p* < 0.001), lower folate concentrations (9.08 ± 8.53 nmol/L) (*p* < 0.001), and lower vitamin B12 concentrations (421.78 ± 289.33 pmol/L) (*p* = 0.005) relative to healthy controls (10.56 ± 3.73 μmol/L, 19.22 ± 7.68 nmol/L and 477.39 ± 200.12 pmol/L, respectively). Table 2 shows the mean scores for each sub-domain in the DLB group. At least one clinically significant symptom (an NPI score of >3 in a given category) was present in 92.7% of individuals, and any symptom (NPI score > 0 in a given category) was present in all individuals. The most frequent clinically significant symptoms were hallucinations (30.4%), apathy (30.4%), and delusions (26.8%) (Table 2 and Figure 2B).

**Table 1:** Comparison of demographic, clinical, and biochemical characteristics of DLB patients versus controls.

Variable	Control (*n* = 134)	DLB (*n* = 82)	*U*	*P-*value
Clinical variables				
Age, mean (SD), y	69.62(5.57)	73.67 (8.10)	7114.50	<0.001***
Sex, male: female	61:73	35:47		
Education, mean (SD), y	8.71 (2.04)	10.18 (4.49)	7069.00	<0.001***
MMSE score, mean (SD)	25.30 (1.13)	15.04 (6.37)	406.50	<0.001***
CDR, mean (SD)	0	1.75 (0.70)		
NPI total scores, mean (SD)	–	27.60 (11.53)		
Disease duration, mean (SD), y	–	2.89 (2.27)		
Biochemical variables				
Plasma tHcy, μmol/L	10.56 (3.73)	17.73 (9.06)	8769.00	<0.001***
Serum Folate, nmol/L	19.22 (7.68)	9.08 (8.53)	1311.50	<0.001***
Serum Vitamin B12, pmol/L	477.39 (200.12)	421.78 (289.33)	4242.00	0.005**

Results are shown as the mean ± SD for the Mann–Whitney *U* test. **Significant difference (*p* < 0.01); ***significant difference (*p* < 0.001); DLB, dementia with Lewy bodies; tHcy, total homocysteine; MMSE, Mini-Mental State Examination.

**Table 2:** Scores of NPI sub-domains in DLB group.

NPI sub-domains	No symptom (NPI = 0)	Mild symptoms (NPI = 1–3)	Clinically significant (NPI > 3)	NPI score mean (SD)
Delusion	45 (54.9)	15 (18.3)	22 (26.8)	2.73 (3.41)
Hallucination	41 (50.0)	16 (19.5)	25 (30.5)	2.82 (3.72)
Agitation/aggression	50 (61.0)	20 (24.4)	12 (14.6)	1.15 (1.99)
Depression/dysphoria	35 (42.7)	34 (41.5)	13 (15.9)	2.76 (2.71)
Anxiety	38 (46.3)	30 (36.6)	14 (17.1)	2.67 (2.74)
Euphoria/elation	77 (93.9)	2 (2.4)	3 (3.7)	0.15 (1.43)
Apathy/indifference	31 (37.8)	26 (31.7)	25 (30.5)	3.35 (3.15)
Disinhibition	65 (79.3)	12 (14.6)	5 (6.1)	0.70 (1.62)
Irritability/lability	38 (46.3)	26 (31.7)	18 (22.0)	2.09 (2.62)
Aberrant motor behavior	40 (48.8)	22 (26.8)	20 (24.4)	2.89 (2.90)
Sleep/nighttime behavior	32 (39.0)	30 (36.6)	20 (24.4)	3.52 (3.06)
Appetite/eating change	44 (53.7)	20 (24.4)	18 (22.0)	1.41 (2.02)

Data are presented as mean (SD) or n (%). DLB, dementia with Lewy bodies; NPI, neuropsychiatric inventory.

### 3.2 Association of elevated tHcy levels with NPI scores

As shown in Table 3, the plasma tHcy levels are significantly associated with total NPI scores in DLB patients (*ρ* = 0.919, *p* < 0.001, Figure 2A). With regards to each NPI sub-domain, a significant association of the plasma tHcy levels was observed in delusion, hallucination, and depression/dysphoria, anxiety, euphoria/elation, apathy/indifference, irritability/lability, aberrant motor behavior, sleep/nighttime behavior, and appetite/eating change (Table 3). However, the association is no longer significant between plasma tHcy levels and the other two sub-domains, such as agitation/aggression and disinhibition (*p* > 0.05).

**Table 3:** Association of plasma tHcy levels with scores of NPI in DLB patients.

NPI sub-domains	DLB patients (*n* = 82)		
	*ρ*	CI	*p*
Delusion	0.365	0.152–0.561	0.001**
Hallucination	0.303	0.076–0.509	0.006**
Agitation/aggression	0.197	−0.005–0.408	0.076
Depression/dysphoria	0.251	0.024–0.460	0.023*
Anxiety	0.256	0.031–0.465	0.020**
Euphoria/elation	0.258	0.052–0.438	0.019*
Apathy/indifference	0.236	0.015–0.453	0.032*
Disinhibition	0.186	−0.040–0.382	0.095
Irritability/lability	0.325	0.110–0.512	0.003**
Aberrant motor behavior	0.325	0.110–0.509	0.003**
Sleep/nighttime behavior	0.321	0.104–0.511	0.003**
Appetite/eating change	0.244	0.012–0.445	0.027*
NPI total score	0.919	0.841–0.966	< 0.001***

ρ, Spearman correlation coefficient; *significant difference (*p* < 0.05); **significant difference (*p* < 0.01); ***significant difference (*p* < 0.001). DLB, dementia with Lewy bodies; NPI, neuropsychiatric inventory.

### 3.3 Association between tHcy levels and duration of dementia, scores of MMSE and CDR

As shown in Table 4, the results of Spearman’s rank correlation test showed the associations between the plasma tHcy level and the duration of dementia, and scores of MMSE and CDR in patients with DLB were not significant (*p* > 0.05) (Table 5).

**Table 4:** Association of serum homocysteine levels with scores of MMSE and CDR and duration of dementia in DLB patients.

	DLB patients (*n* = 82)		
	*ρ*	CI	*p*
Duration of dementia	−0.002	−0.237 – 0.242	0.988
MMSE	0.119	−0.111 – 0.321	0.285
CDR	0.025	−0.192 – 0.232	0.824

**Table 5:** Frequency of different BPSD in DLB patients.

	No symptom (0)	Mild symptoms (1–3)	Clinically significant (>3)
Delusions	45(54.9)	15(18.3)	22(26.8)
Hallucinations	41(50.0)	16(19.5)	25(30.5)
Aggression/Agitation	50(61.0)	20(24.4)	12(14.6)
Depression/Dysphoria	35(42.7)	34(41.5)	13(15.9)
Anxiety	38(46.3)	30(36.6)	14(17.1)
Elation/Euphoria	77(93.9)	2(2.4)	3(3.7)
Apathy	31(37.8)	26(31.7)	25(30.5)
Disinhibition	65(79.3)	12(14.6)	5(6.1)
Irritability	38(46.3)	26(31.7)	18(22.0)
Aberrant motor behavior	40(48.8)	22(26.8)	20(24.4)
Sleep and night-time behavior changes	32(39.0)	30(36.6)	20(24.4)
Appetite and eating abnormalities	44(53.7)	20(24.4)	18(22.0)

DLB, dementia with Lewy bodies; data are presented as *n* (%).

## 4 Discussion

Our study suggests that DLB patients had a higher risk of BPSD relative to the control group, particularly in terms of hallucinations, delusions, and apathy, and a lower risk of exultation/euphoria. Our findings are partially consistent with previous reports (Schwertner et al., 2022), which observed a higher risk of hallucinations and delusions and a lower risk of exaltation/pleasure in DLB patients. Treatment of DLB is focused on the BPSD, besides the cognitive, motor, and other non-motor symptoms that represent the core or most common features of the disorder. However, particular interventions and management of BPSD remain limited. Based on the present study’s findings, we can conclude that high levels of tHcy may be a risk factor for BPSD in DLB. We have demonstrated in previous studies (Zhang et al., 2021) that elevated plasma tHcy levels are independently associated with DLB and that BPSD is a core symptom in DLB patients. Therefore, the current study supported the hypothesis that future randomized clinical trials with tHcy-reducing therapies should be conducted to provide further evidence of the relationships between tHcy and BPSD of DLB.

In this study, DLB patients also had higher tHcy levels than the control group (*p* < 0.01). Also, we observed that elevated plasma tHcy levels were significantly associated with the total NPI score based on the “caregivers” report. In particular, a significant association was found with the following NPI subdomains: delusion, hallucination, depression/dysphoria, anxiety, euphoria/elation, apathy/indifference, irritability/lability, aberrant motor behavior, sleep/nighttime behavior, appetite/eating change. However, there was no association between the plasma tHcy level MMSE and CDR scores in DLB patients.

The associations between homocysteine levels and cognitive functions in vascular dementia, AD, and PD have been extensively studied in the past (Clarke et al., 1991; Ma et al., 2017; Ravaglia et al., 2005; Ravaglia et al., 2000; Seshadri et al., 2002). Additionally, our previous studies have shown that caregiver burden increases and psychological status deteriorates, particularly in patients with AD, DLB, and MCI. However, studies on the relationship between homocysteine levels and behavioral and psychological symptoms of dementia (BPSD) are limited. To date, no studies have reported the association between homocysteine levels and BPSD in DLB patients.

The results of this case–control study demonstrate that elevated plasma tHcy levels are significantly associated with the total NPI score in Chinese DLB patients. A significant correlation was also found between elevated plasma tHcy levels and 10 NPI sub-domains, including delusions, hallucinations, depression/dysphoria, anxiety, euphoria/elation, apathy/indifference, irritability/lability, aberrant motor behavior, sleep/nighttime behavior, appetite/eating changes.

The results of our study are inconsistent with a previous study involving 27 patients without BPSD and 23 control patients with BPSD, which reported that high homocysteine levels were not significantly associated with BPSD in AD patients (Tabet et al., 2006).

However, other studies have shown that high homocysteine levels are significantly associated with BPSD in AD patients, indicating that increased plasma homocysteine can exacerbate cognitive dysfunction in AD patients (Kim and Lee, 2014; Soni et al., 2019; Zheng et al., 2014). In addition, some previous studies in patients with schizophrenia, psychosis, or obsessive-compulsive disorder reported similar findings, showing a relationship between elevated plasma tHcy levels and a higher risk of depression, schizophrenia, and other psychiatric disorders (Monji et al., 2005; Reif et al., 2005; Vogel et al., 2009).

Furthermore, our findings are partially consistent with the results of Kim and Lee (2014) and Soni et al. (2019), who observed a significant correlation between elevated plasma tHcy level and the total NPI score, as well as the following NPI sub-domains in AD patients: delusion, agitation/aggression, depression/dysphoria, elation/euphoria, apathy/indifference, and disinhibition.

In our study, DLB patients exhibited more pronounced and frequent BPSD than AD patients, which may explain the association of elevated plasma tHcy levels with more NPI sub-domains in DLB patients than in AD patients in the studies by Kim et al. and Soni et al. (2019).

In addition, the heterogeneity of the BPSD spectrum might account for the differences in the association between elevated plasma tHcy levels and sub-domains such as agitation/aggression and disinhibition, which were observed in AD patients of Kim et al. study but not in the DLB patients in our study. The findings of this study indicate that elevated plasma tHcy levels are significantly associated with BPSD in DLB patients, particularly in 10 NPI sub-domains, excluding agitation/aggression and disinhibition.

Managing BPSD in DLB is challenging, and no medications are specifically licensed for treating these symptoms. Many of the medications used can carry significant risks for patients. Therefore, identifying whether these symptoms are related to elevated homocysteine levels is crucial.

This study shows an association between elevated homocysteine levels and BPSD, along with its sub-domains. These results might support the hypothesis that controlling homocysteine levels could be an effective therapeutic approach for managing BPSD. Further research including prospective follow-up studies and randomized controlled trials, is needed to determine whether high homocysteine levels are a cause, rather than a consequence, of BPSD.

We did not find any significant correlation between plasma tHcy levels, MMSE scores, or the duration of dementia in DLB patients. This lack of a significant correlation may be due to the small sample size or the fact that MMSE is a brief screening tool that does not thoroughly assess the core clinical features commonly affected in DLB. Previously, the overall results show that an increased tHcy concentration is correlated with decreased cognitive functions, although the findings of the previous studies were inconsistent (Ma et al., 2017; Zhang et al., 2021). However, the results of the present study showed no association between tHcy levels and MMSE scores in DLB patients, which is consistent with the findings of an AD study by Tabet et al. (2006). We speculated that such findings may be because visuospatial and attention/executive functions tend to be more impaired in DLB patients. However, MMSE screens for performance in these domains are brief and may not highlight deficits.

Additionally, earlier BPSDs tend to be more prominent than cognitive impairment, at least early on in DLB. A possible explanation regarding no association of the tHcy level with the duration of DLB dementia is that an elevated tHcy level precedes or appears at an early stage of cognitive impairment. The findings of this study suggest that plasma tHcy level is not associated with cognitive impairment and the duration of dementia in DLB patients. In the future, other research will expand the sample size, and a longitudinal study will be conducted to investigate the related factors of BPSD in DLB patients.

Further elucidation of the underlying mechanisms regarding the observed associations between elevated plasma tHcy levels and BPSD of DLB patients is needed. However, various hypotheses have been suggested to explain the correlation between hyperhomocysteinemia and psychiatric disorders. First, homocysteine may directly activate *N*-methyl-d-aspartate (NMDA) receptors or convert into homocysteine acid, a highly potent neurotoxic metabolite, an NMDA receptor agonist, which results in cell death and excessive intracellular calcium influx to exert excitotoxic damage to neurons (Bao et al., 2022; Lehmann et al., 1988). Secondly, according to the vascular depression hypothesis (Kim and Pae, 1996), the high level of homocysteine could cause direct damage and chronic inflammatory responses in vascular endothelial cells, which in turn exacerbates DLB and contributes to “vascular depression” due to vascular pathology. Finally, a previous study has suggested that apathy in AD patients is associated with dopaminergic system dysfunction (Folstein et al., 1975). Then, we speculated that high homocysteine levels could inhibit one-carbon metabolism, which is involved in dopamine formation, resulting in BPSD in DLB patients. This suggests that homocysteine could impair neuronal plasticity and promote neuronal degeneration to contribute to the pathogenesis of neurodegenerative and psychiatric symptoms in DLB. Therefore, we theorize that the high levels of homocysteine possibly play a role in the BPSD of DLB by the above several mechanisms working together due to the complex pathology of DLB. Based on our various other results suggesting that homocysteine levels are correlated with not only AD and DLB but also different types of dementia, we believe that the association also needs to be explored in frontotemporal dementia, in which behavioral abnormalities are prevalent.

### 4.1 Limitations

There are several limitations to our study. First, as a cross-sectional and retrospective study, the inferences drawn from this study are limited. Our findings must be confirmed through longitudinal studies. Second, the sample consisted only of individuals of Chinese ethnicity, and the sample size was not large. Third, MMSE is only a brief screening that assesses only a few cognitive domains.

A comprehensive neuropsychological assessment should be conducted in future studies to provide a more in-depth evaluation of cognitive domains and core clinical symptoms, as this may reveal impairments. In addition, the NPI is an informant-based assessment, relying on information provided by the patient’s caregiver rather than a clinician. Moreover, no patient self-reported screeners for depression, anxiety, or other conditions were administered.

## 5 Conclusion

We found that nearly all patients with dementia with (DLB) experienced behavioral and psychological symptoms of dementia (BPSD), with the most frequent being hallucinations, apathy, and delusions. Our study is the first to suggest that the presence of BPSD in DLB patients may be associated with elevated plasma tHcy levels, although a causal relationship has not been confirmed.

We found that almost all patients with dementia with Lewy bodies (DLB) experienced behavioral and psychological symptoms of dementia (BPSD), with the most frequent being hallucinations, apathy, and delusions. Our study is the first to suggest that the presence of BPSD in DLB patients may be associated with elevated plasma tHcy levels, although a causal relationship has not been confirmed. It is hypothesized that elevated homocysteine levels may play a role in the development of BPSD in DLB, but the underlying pathophysiological mechanisms remain to be elucidated. Future research with larger sample sizes and longitudinal studies will be needed to investigate the factors associated with BPSD in DLB patients.

## Data Availability

The datasets presented in this study can be found in online repositories. The names of the repository/repositories and accession number(s) can be found in the article/supplementary material.
